# Research Progress on Polyurethane-Based Grouting Materials: Modification Technologies, Performance Characterization, and Engineering Applications

**DOI:** 10.3390/polym17172313

**Published:** 2025-08-27

**Authors:** Langtian Qin, Dingtao Kou, Xiao Jiang, Shaoshuai Yang, Ning Hou, Feng Huang

**Affiliations:** 1 School of Engineering and Technology, China University of Geosciences Beijing, Beijing 100083, China; qqlltt321@163.com (L.Q.); yangshaoshuai2001@163.com (S.Y.); sdhouning@email.cugb.edu.cn (N.H.); 2 School of Emergency Management and Safety Engineering, China University of Mining and Technology, Beijing 100083, China; kdtcumt@126.com; 3 Beijing Rail Transit Construction Management, Co., Ltd., Beijing 100068, China; 4 China Railway 23rd Bureau Group Co., Ltd., Chengdu 610072, China; jiangxiao1907@163.com

**Keywords:** polyurethane, grouting materials, modification technology, performance characterization, engineering applications

## Abstract

Polyurethane grouting materials are polymer materials formed through the reaction of polyisocyanates and polyols. They play important roles in underground engineering, tunnel construction, and mining due to their fast reaction rate, high bonding strength, and excellent impermeability. However, traditional polyurethane grouting materials have shortcomings such as high reaction heat release, high brittleness, and poor flame retardancy, which limit their applications in high-demand engineering projects. This paper systematically reviews the research progress on modified polyurethane grouting materials. Four major modification technologies are summarized: temperature reduction modification, flame retardant modification, mechanical enhancement, and environmental adaptability improvement. A multi-dimensional performance characterization system is established, covering slurry properties, solidified body performance, microstructure characteristics, thermal properties and flame retardancy, diffusion grouting performance, and environmental adaptability. The application effects of modified polyurethane grouting materials in grouting reinforcement, grouting water plugging, and grouting lifting are analyzed. Future development directions are projected. This review is particularly valuable for researchers and engineers working in tunneling, mining, geotechnical engineering, and infrastructure rehabilitation.

## 1. Introduction

With the continuous expansion of modern engineering construction scales and increasingly complex construction environments [1,2], issues related to reinforcement, seepage control, and repair in engineering projects have become increasingly prominent. Grouting refers to an engineering technique that injects grout into formation pores and cracks under certain pressure, achieving reinforcement, seepage control, and repair through grout solidification and hardening [3]. In recent years, this technique has been widely applied in numerous fields such as underground engineering, tunnel construction, and mining engineering [4,5]. This technology can effectively improve the physical and mechanical properties of the injected medium and enhance the stability and durability of engineering structures [6].

As a key component of grouting technology, grouting materials directly determine the grouting effectiveness and engineering quality [7]. Based on chemical composition, grouting materials can be classified into two major categories: inorganic grouting materials and organic grouting materials. Inorganic grouting materials mainly include cement–clay grouting materials, cement–water glass grouting materials, and inorganic silicate grouting materials [8]; organic grouting materials mainly include acrylate grouting materials [9,10], acrylamide grouting materials [11], lignin-based grouting materials [12], urea-formaldehyde resin grouting materials [13], epoxy resin grouting materials [14], and polyurethane grouting materials, among others. Among numerous organic grouting materials, polyurethane grouting materials have become one of the most widely used and rapidly developing grouting materials due to their moderate viscosity, controllable curing time, resistance to wear and chemical corrosion, good bonding properties, and high mechanical strength [15,16].

Polyurethane (PU) grouting materials are macromolecular materials formed through nucleophilic addition reactions using polyisocyanates and polyols as the main raw materials [17,18,19,20]. Since Bayer first synthesized PU materials through addition polymerization of polyols and polyisocyanates in 1937 [21], polyurethane technology has undergone significant development. Early industrial applications focused on rigid foams [22] and elastomers [23], while the understanding of microphase separation structure [24] and urethane bond formation mechanisms [25] provided a theoretical foundation for modern polyurethane material design [26]. The development of waterborne polyurethane systems [27] and advanced catalyst technologies [28] further expanded the application scope of polyurethane materials in construction and engineering fields [29]. Although polyurethane grouting materials possess numerous advantages, single polyurethane grouting materials still exhibit problems such as high reaction heat release, high brittleness, insufficient temperature resistance, and poor flame retardancy in complex engineering environments, limiting their applications in high-demand engineering projects.

**Figure 1:**
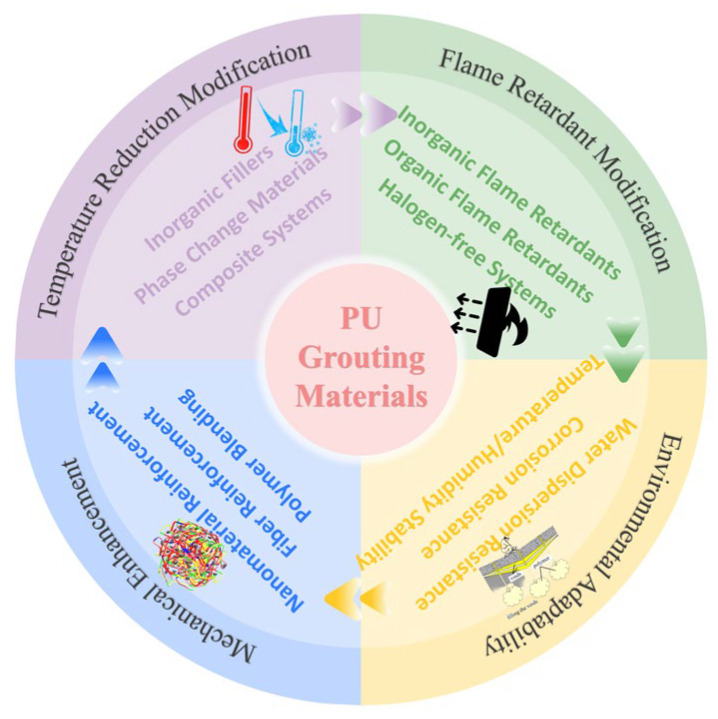
Research Framework for Modified Polyurethane Grouting Materials.

To gain a deeper understanding of the modification mechanisms and engineering application effects of polyurethane-based composite grouting materials and promote their widespread application under complex working conditions, this paper first systematically classifies polyurethane grouting materials, explores the research progress in four major modification technologies including temperature reduction modification, flame retardant modification, mechanical property enhancement, and environmental adaptability improvement, summarizes the latest understanding of material performance characterization systems and formation mechanisms, analyzes their typical applications in grouting reinforcement, grouting water plugging, and grouting repair, and provides prospects for future efficient modification and industrial application of polyurethane-based composite grouting materials, as illustrated in Figure 1.

## 2. Fundamental Principles and Classification of Polyurethane Grouting Materials

### 2.1. Basic Reaction Principles and Chemical Structure

**Figure 2:**
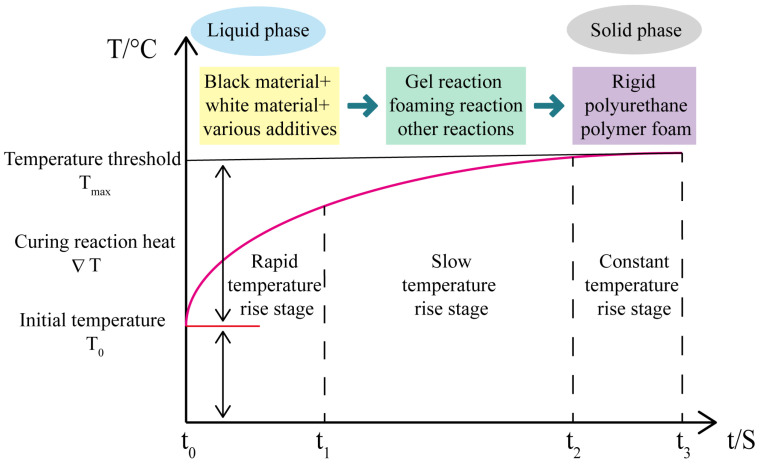
Temperature Rise Mechanism Diagram of Polyurethane Curing Process. Redrawn from ref. [31].

Polyurethane grouting materials are formed through nucleophilic addition reactions between isocyanate groups (-NCO) and hydroxyl-containing compounds (-OH). The expansion and curing process of polyurethane grouting materials mainly involves two chemical reactions: gel reaction and foaming reaction [30]. In the gel reaction, isocyanate reacts with polyols to generate the polyurethane main chain, forming urethane bonds (-NHCOO-); in the foaming reaction, isocyanate combines with water under catalysis to produce carbon dioxide gas, causing volume expansion of the slurry. The temperature evolution during these reaction processes is characterized by three distinct stages, as shown in Figure 2.

**Figure 3:**
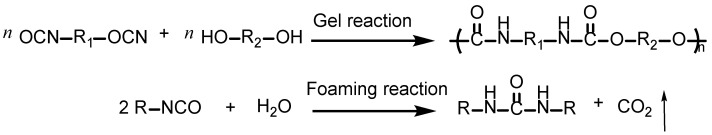
Primary chemical reaction processes of polyurethane grouting materials.

These two reaction processes work together to determine the foaming characteristics, mechanical properties, and filling capacity of polyurethane grouting materials, as shown in Figure 3.

Isocyanate (-NCO) serves as an essential raw material for polyurethane formation, primarily constituting the hard segment structure and acting as the main contributor to the mechanical strength of the material [32,33], while providing structural stability and thermal stability through physical crosslinking [34]. The most widely used polyisocyanates in industry mainly include 4,4′-diphenylmethane diisocyanate (MDI) and toluene diisocyanate (TDI). MDI has a molecular formula of C_15_H_10_N_2_O_2_, and its rigid aromatic structure connecting two benzene rings through a methylene bridge gives it moderate reactivity and good thermal stability, making it the most widely used in grouting materials. TDI includes two forms: 2,4-isomer and 2,6-isomer, with a molecular formula of C_9_H_6_N_2_O_2_. The reactivity of the isocyanate group at the 4-position in 2,4-TDI is significantly higher than that at the 2-position, and this reactivity difference gives TDI better reaction controllability. However, due to its relatively high volatility and toxicity, its application in grouting materials is relatively limited.

Polyols (-OH) mainly form soft segment structures in polyurethane grouting materials, endowing the materials with flexibility and elasticity, and determining the low-temperature performance and deformation ability of the materials [32,35]. Polyols are mainly divided into two categories: polyether polyols and polyester polyols. Polyether polyols are typically represented by polypropylene glycol (PPG), with molecular main chains connected by ether bonds, possessing good flexibility, low-temperature performance, and hydrolysis resistance. Polyester polyols are formed by polycondensation of dibasic acids and diols, with main chains containing ester bonds. Compared to polyether polyols, they have higher cohesive energy and better mechanical strength, but are prone to hydrolysis in humid environments. The molecular weight and functionality of polyols directly affect the crosslinking density and mechanical properties of polyurethane materials, and the hardness and elasticity of the materials can be precisely controlled by adjusting the ratio of polyols with different molecular weights. The molecular structures and chemical formulas of the main raw materials are summarized in Table 1.

In addition to the two main reactive components of isocyanate and polyols, polyurethane grouting materials may contain functional additives such as catalysts and surfactants to regulate the reaction process and material properties. Foaming relies on CO_2_ from isocyanate–water reactions. Catalysts are mainly divided into organometallic catalysts that promote gel reactions and tertiary amine catalysts that promote foaming reactions, and the ratio of these two directly affects the foaming speed and curing time of the material [36]. For specialized applications, blowing agents can be added. Blowing agents are divided into chemical blowing agents and physical blowing agents, which achieve material expansion through gas generation or evaporative heat absorption. Surfactants can stabilize the bubbles generated during the foaming process, ensuring the formation of uniform cellular structures, while improving the wetting properties and penetration capacity of the slurry. The types and dosages of these functional additives can be selectively added according to specific application requirements.

### 2.2. Classification of Polyurethane Grouting Materials

Polyurethane grouting materials can be classified from multiple dimensions based on different technical characteristics and application requirements, as shown in Table 2. From the perspective of engineering applications, the most important classification methods include component systems, water reaction characteristics, density characteristics, and functional uses. These classification methods intersect with each other and collectively constitute the technical system of polyurethane grouting materials.

Classification by component system is the most fundamental classification method for polyurethane grouting materials, which directly determines the construction method and reaction mechanism of the materials. Single-component polyurethane grouting materials adopt prepolymer technology, where isocyanate is pre-reacted with part of the polyols to prepare prepolymers containing active NCO groups, which react and cure when encountering moisture in the environment. Wang et al. [37] developed single-component high-strength polyurethane grouting materials that achieve optimal compressive strength and a suitable gel time when the NCO group content is approximately 20%. Single-component materials are convenient for construction and require no on-site proportioning and mixing, making them particularly suitable for emergency rescue applications. However, their performance adjustment range is relatively limited, and the curing time mainly depends on environmental humidity. Two-component materials react after mixing the A and B components in proportion. Huang et al. [38] found that the mass ratio of soft and hard polyether polyols is a key factor affecting material performance. Two-component systems can precisely control key parameters such as curing time and foaming ratio by changing proportions and additives, offering a wider range of applications.

From the perspective of water reaction characteristics, different types of materials exhibit distinctly different response behaviors to water environments. Wang et al. [15] classified materials into hydrophilic, hydrophobic, and solvent-free types based on the response characteristics of cured bodies to water. Hydrophilic materials introduce hydrophilic groups such as ionic groups and polyethylene oxide segments, react strongly with water and expand rapidly, with expansion ratios reaching more than six times, providing unique advantages in handling large water inrush projects. Hydrophobic materials adopt hydrophobic molecular structures, react mildly with water, and exhibit excellent water resistance after curing, making them suitable for long-term immersion environments [39]. Solvent-free polyurethane grouting materials contain no volatile organic compounds, have 100% solid content, and feature low curing shrinkage and good environmental compatibility, conforming to the development trend of green building materials [40].

Classification by density characteristics mainly considers the cellular structure and mechanical performance requirements of materials. Foaming materials typically have densities between 0.1 and 0.8 g/cm^3^, possess porous structures, exhibit significant volume expansion during the reaction, can fully fill irregular cracks and cavities, and provide certain thermal insulation effects, and are mainly used for large-volume void filling and water sealing [41]. Elastomer materials have densities between 1.0 and 1.4 g/cm^3^, and although their foaming ratios are smaller, they feature high strength and high modulus load-bearing requirements [42].

From the perspective of functional applications, different types of materials are specifically optimized for particular engineering requirements. Water-blocking materials focus on impermeability and stability in dynamic water environments; reinforcement materials emphasize mechanical strength and adhesive properties; lifting materials require controllable expansion force and precise volume control; special environment materials, through special molecular design, can maintain stable performance in extreme environments such as marine engineering [43], high temperature, and chemical corrosion [44].

**Table 2:** Classification and characteristics of polyurethane grouting materials.

Classification Dimension	Type	Main Characteristics	Typical Applications
Component System	Single-component [38]	Prepolymer type, moisture curing	Emergency water blocking, road repair
	Two-component [38]	A/B component mixing reaction, adjustable performance	Precision grouting, structural reinforcement
Water Reaction Characteristics	Hydrophilic [15]	Rapid expansion, high expansion ratio	Large water inrush blocking projects
	Hydrophobic [15]	Mild reaction, low water absorption	Long-term immersion environment
	Solvent-free [15]	100% solid content, environmentally friendly	Projects with strict environmental requirements
Density Characteristics	Foaming type [42]	0.1–0.8 g/cm^3^, porous structure, high expansion ratio	Large void filling, thermal insulation, and emergency water blocking
	Elastomer type [44]	1.0–1.4 g/cm^3^, dense structure, high strength	Structural reinforcement, load-bearing applications, and infrastructure rehabilitation
Functional Applications [15]	Water blocking type	Excellent impermeability, rapid sealing	Seepage prevention and plugging
	Reinforcement type	High strength, strong adhesion	Soil and rock mass reinforcement
	Lifting type	Controllable expansion force, precise volume control	Settlement structure repair
	Special environment type [43]	Corrosion resistance, high temperature resistance, and other special properties	Marine engineering, chemical environments

## 3. Modification Technologies of Polyurethane Grouting Materials

Modified polyurethane grouting materials are novel composite material systems formed by introducing functional components, such as inorganic fillers, organic polymers, and nanomaterials, through physical blending, chemical modification, in situ polymerization, and other methods, while maintaining the excellent properties of the polyurethane matrix. These modification technologies not only effectively reduce the reaction temperature and fire risk of the materials but also significantly improve their mechanical properties, durability, and environmental adaptability. According to different modification purposes and technical routes, the modification technologies of polyurethane grouting materials can be divided into four major categories: temperature reduction modification, flame retardant modification, mechanical enhancement modification, and environmental adaptability improvement.

### 3.1. Temperature Reduction Modification Technologies

Polyurethane grouting materials undergo intense exothermic reactions during the curing process, with maximum reaction temperatures reaching above 140 °C [45]. This not only poses fire safety hazards but also causes thermal stress within the material, affecting its mechanical properties and durability. Especially in mining engineering, excessively high reaction temperatures may trigger serious safety accidents such as gas explosions [46]. Therefore, reducing the reaction temperature of polyurethane grouting materials is an important direction in modification research.

#### 3.1.1. Temperature Reduction by Inorganic Fillers

**Figure 4:**
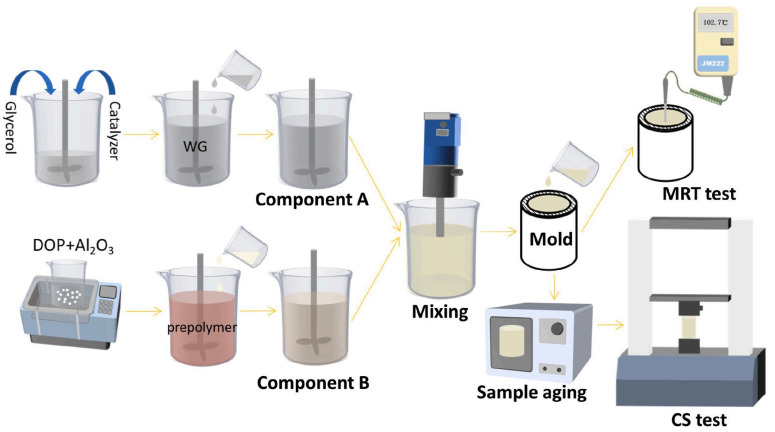
Typical preparation process of inorganic filler-modified polyurethane grouting materials. Reprinted with permission from ref. [49].

Temperature reduction by inorganic fillers is achieved by incorporating inorganic fillers with high thermal conductivity or high heat capacity into the polyurethane system, utilizing their physical heat absorption and thermal conduction effects to reduce the maximum temperature of the reaction system. Qin Chuanrui et al. [47] prepared polyurethane composites using nano fly ash as aggregate through the in situ polymerization method. By leveraging the thermal conduction heat absorption effects of fly ash, the maximum curing temperature was reduced to 82 °C with a compressive strength of 44 MPa. Liu Yang et al. [48] used surface-modified coal gangue powder (MCG) as filler and improved the filler–matrix interface compatibility, reducing the maximum reaction temperature of MCG/PU composites from 138.3 °C to 116.8 °C while maintaining the compressive strength at 51.8 MPa and achieving curing within approximately 2 min. Xiaofeng Yu et al. [49] introduced thermally conductive aluminum oxide (Al_2_O_3_) into the water glass/polyurethane (WG/PU) system. Through accelerating heat dissipation and regulating reaction kinetics, the maximum reaction temperature of the composite was reduced by 14.9 °C (12.7%), while the compressive strength increased by 13.84 MPa (27.3%), achieving the dual effects of temperature reduction and mechanical enhancement, and the preparation process is shown in Figure 4.

#### 3.1.2. Temperature Reduction by Phase Change Materials

**Figure 5:**
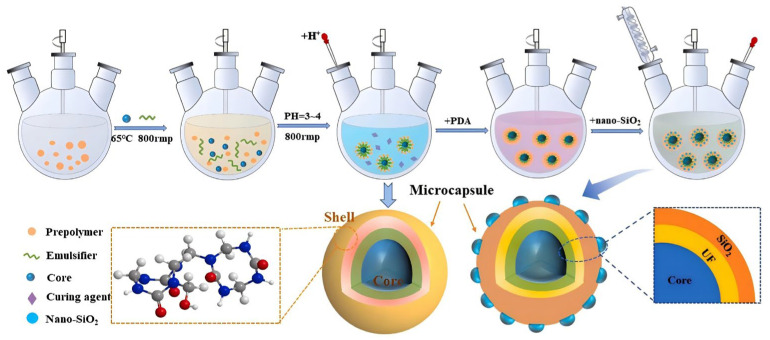
Preparation process of phase change microcapsules for temperature reduction. Reprinted with permission from ref. [51].

Temperature reduction by phase change materials is achieved by introducing phase change materials into the polyurethane system and utilizing their heat absorption effect during the phase change process to regulate the exothermic behavior of the curing reaction. Fan Kangxin et al. [50] prepared a series of microencapsulated phase change materials (MEPCMs) with polyurethane shells and paraffin cores through interfacial polymerization, and then blended them with polyols and catalysts to react with polyisocyanates to prepare polyurethane grouting materials. The maximum curing reaction temperature was 98.7 °C, the curing time was 235 s, and the compressive and tensile strengths of the solidified body were 44.9 MPa and 14.7 MPa, respectively. While maintaining good slurry injectability, solidified body strength, and thermal stability, it achieved regulation of the curing exothermic behavior. Shangxiao Liu et al. [51] prepared silica-based urea-formaldehyde heteroshell phase change microcapsules (S-MPCM) containing polydopamine, and fabricated composite polyurethane foam materials by incorporating 10% S-MPCM. The modifying effect of polydopamine improved the compatibility between microcapsules and polyurethane matrix, with the microcapsule encapsulation rate reaching 76.5%, melting enthalpy of 162.4 J/g, and the maximum internal temperature of composite foam reduced to 53.7 °C, showing excellent thermal regulation performance. The preparation process is shown in Figure 5.

#### 3.1.3. Composite Temperature Reduction Systems

Composite temperature reduction systems achieve better cooling effects through the synergistic action of multiple temperature reduction mechanisms. Han Xiangyu et al. [52] and Cheng Yulong et al. [53] both adopted the composite modification strategy of expanded graphite (EG)/dimethyl methylphosphonate (DMMP). The research found that when the addition amounts of EG and DMMP were both 1 wt%, the maximum reaction temperature of polyurethane grouting materials could be reduced by 36.1%, and the viscosity was reduced by 33.2%. This composite system not only achieved significant temperature reduction but also improved the fluidity and flame-retardant properties of the material, effectively solving the “thermal runaway” hazard of grouting materials. Mei Fanghua et al. [54] adopted the composite modification method of epoxy resin prepolymer and water glass. By preparing prepolymers through the reaction of epoxy resin and isocyanate and combining with the inorganic filler effect of water glass, they achieved the dual objectives of temperature reduction and mechanical enhancement. The maximum reaction temperature of the modified polyurethane grouting material was reduced to 89 °C, the compressive strength was improved to 0.27 MPa, and the modified foam cell size became smaller and more regular.

### 3.2. Flame Retardant Modification Technologies

Polyurethane grouting materials typically have an oxygen index of only 16–18%, making them highly flammable and prone to ignition under high temperature or open flame conditions, which poses serious fire safety hazards [55]. Unlike temperature reduction modification, which primarily prevents thermal runaway by controlling reaction temperature, flame retardant modification technologies focus on preventing material combustion at the source and enhancing the inherent fire resistance of the material itself. Particularly in mining and other underground engineering applications, insufficient flame retardancy may trigger major safety accidents. Even when reaction temperatures are controlled, external ignition sources can still ignite the material and cause rapid flame spread. Therefore, improving the flame-retardant performance of polyurethane grouting materials from the source through chemical or physical modification methods is a key technology to ensure their safe application in complex engineering environments [56].

#### 3.2.1. Inorganic Flame Retardant Modification

**Figure 6:**
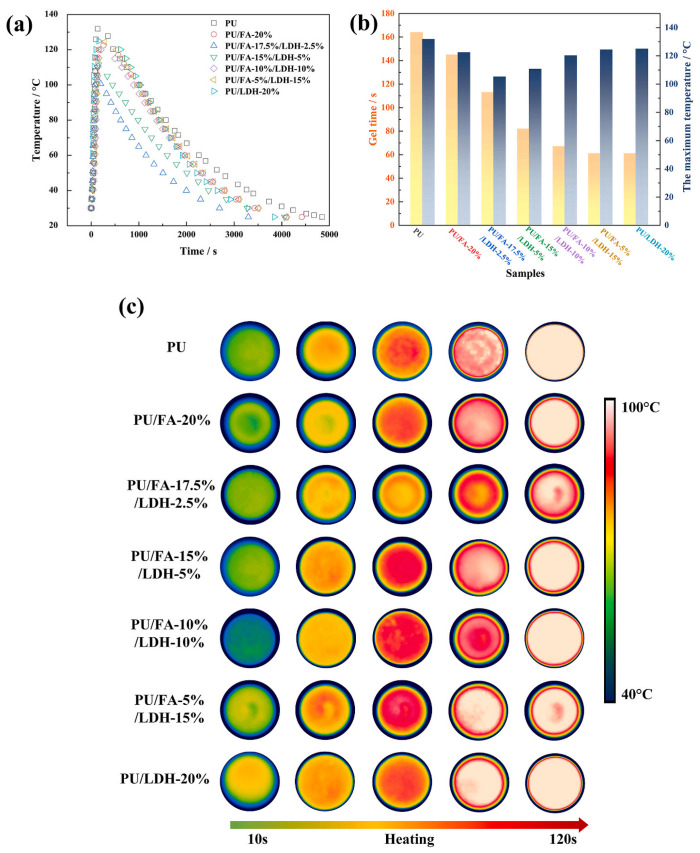
Flame retardant performance of inorganic filler-modified polyurethane grouting materials. Reprinted with permission from ref. [59].

Inorganic flame retardant modification is achieved by adding inorganic flame retardants or compositing with inorganic materials, utilizing their non-combustible properties, endothermic decomposition, and combustible gas dilution mechanism to achieve flame retardant effects. Cui Yaping et al. [57] modified polyurethane composite materials with water glass and found that water glass not only improved flame retardant performance but also served as a reinforcing agent to enhance mechanical properties. When the dosage was 5 g, the tensile strength and compressive strength reached 0.280 MPa and 0.068 MPa, respectively. Zhang Sitong et al. [58] used surface-modified fly ash as a partial replacement for conventional fillers to prepare PU/FA composite materials, achieving flame retardant modification while reducing production costs and environmental pollution. The research found that surface-modified fly ash improved the compatibility between hard and soft segments of polyurethane, giving the composite materials enhanced thermal stability, high hydrophobicity, and flame-retardant properties. Building on this foundation, Sitong Zhang et al. [59] further employed nano-layered double hydroxide (LDH) combined with fly ash for composite modification. The research revealed that adding 2.5% LDH not only reduced the maximum reaction temperature by 26.7 °C but also significantly improved the flame retardant performance of the material. Figure 6 presents comprehensive evidence for these improvements: (a) temperature–time curves during the curing process of different PU/FA/LDH compositions, (b) the relationship between gel time and maximum temperature for various formulations, and (c) surface IR thermography images showing heat distribution patterns during curing, where the cooler surface temperatures with LDH addition demonstrate enhanced heat dissipation effects.

#### 3.2.2. Organic Flame Retardant Modification

Organic flame retardant modification is primarily achieved by adding organic flame retardants containing phosphorus, nitrogen, and other elements, utilizing their charring, endothermic decomposition, and free radical scavenging mechanisms during combustion to achieve flame retardancy. Jia Ben et al. [60] synthesized N-P synergistic halogen-free flame retardant polyphosphoric acid piperazine and applied it to polyurethane grouting materials. When the addition amount was 20%, the material LOI reached 28.5%, the UL-94 rating reached the V-0 level, and the compressive strength remained above 40 MPa. After combustion, a dense char layer was formed, exhibiting typical condensed-phase flame-retardant characteristics. Chen Xingming et al. [61] addressed the high halogen content problem in conventional polyurethane grouting reinforcement materials by synthesizing a reactive halogen-free organic grouting reinforcement material (PU-NH). Compared with ordinary PU, smoke release was reduced by 39.3%, carbon monoxide release was reduced by 50.75%, and the dense char layer formed after combustion could effectively isolate oxygen and reduce the heat conduction rate. Building on this foundation, Haiyan Wang et al. [62] further developed a low heat accumulation flame-retardant polyurethane grouting material through a composite strategy of adding nano-scale flame retardants and phosphorus-based flame retardants. The material synthesis reaction temperature was reduced to 51.2 °C, the thermal decomposition onset temperature was increased to 286 °C, and the flame retardant performance was significantly improved, while greatly reducing the heat accumulation effect on surrounding coal during the synthesis process.

### 3.3. Mechanical Property Enhancement Technologies

Although polyurethane grouting materials possess excellent bonding performance and rapid curing characteristics, their solidified bodies often suffer from high brittleness, insufficient toughness, and low compressive strength, making it difficult to meet the load-bearing requirements under complex working conditions [63]. Different density grades of polyurethane grouting materials face distinct mechanical performance challenges: foamed materials need to improve load-bearing capacity while maintaining lightweight characteristics, while elastomer-type materials require further enhancement in toughness and impact resistance [64]. These mechanical performance deficiencies limit the application scope of polyurethane grouting materials in high-demand engineering projects. Therefore, significantly improving the mechanical properties of polyurethane grouting materials through the introduction of reinforcing materials or modification technologies has become an important research direction for expanding their engineering applications [65].

#### 3.3.1. Nanomaterial Reinforcement

Nanomaterials can significantly improve the mechanical properties of polyurethane at relatively low addition amounts due to their unique size effects and surface effects. Nano-SiO_2_ reinforcement is the most widely used nanomaterial modification technology, with its reinforcement mechanisms primarily including filling effects, interfacial effects, and network structure regulation. Jia Xiaopan et al. [66] prepared modified polyurethane grouting materials by incorporating different contents of nano-SiO_2_ through the in situ polymerization method. When the nano-SiO_2_ content was 6%, the sample compressive strength reached a maximum value of 0.115 MPa. SEM analysis showed that appropriate amounts of nano-SiO_2_ significantly reduced foam cell size and improved distribution uniformity, but excessive addition led to local agglomeration. Shi Li et al. [67] prepared polyurethane grouting materials through the prepolymer method, and results showed that surface hydroxyl groups of nano-SiO_2_ reacted with NCO groups to form chemical bonds. At a content of 0.6%, the compressive strength reached a maximum value of 0.118 MPa. Li Yadi et al. [68] prepared nano-silica uniformly dispersed in polyurethane through an in situ method, which combined with the polyurethane network through chemical bonds and significantly improved the mechanical properties of the solidified body. Underground water-blocking pilot engineering tests confirmed that the material had high practical water-blocking efficiency. Graphene also demonstrated excellent effects in polyurethane modification. Zhou Xinxing et al. [69] employed graphene and thermoplastic polyurethane for composite modification of polyurethane grouting materials. The addition of graphene increased the compressive strength by more than 100%, reaching 15.0–43.8 MPa, and the material exhibited good strength, durability, and elasticity.

#### 3.3.2. Fiber Reinforcement

**Figure 7:**
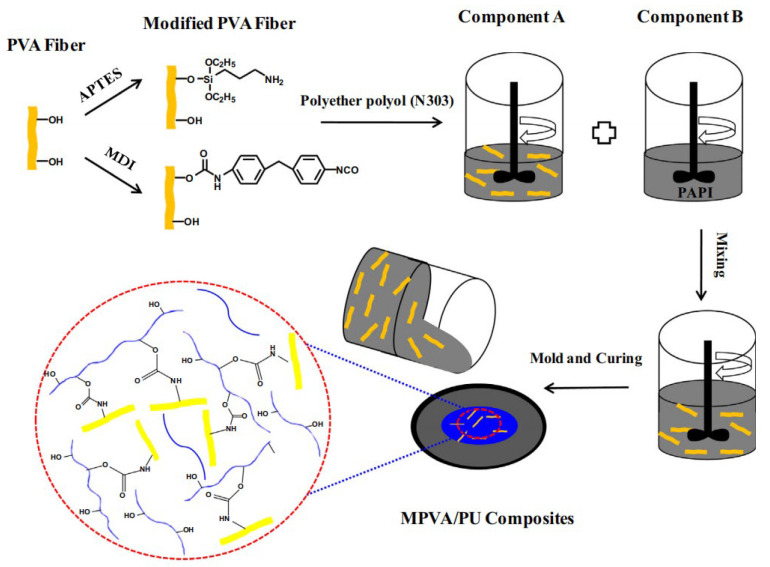
Preparation process of fiber-reinforced polyurethane grouting materials. Reprinted with permission from ref. [70].

Fiber reinforcement improves the toughness and crack resistance of materials by adding various fibers to the polyurethane matrix, utilizing the high strength and bridging action of fibers. The reinforcement mechanism is primarily based on stress transfer and crack bridging, achieving load transfer and delaying crack propagation through effective bonding at the fiber–matrix interface. Ming Cao et al. [70] prepared modified polyvinyl alcohol fiber-filled polyurethane grouting materials by surface modification of PVA fibers with γ-aminopropyltriethoxysilane (APTES) and diphenylmethane diisocyanate (MDI), with the preparation process shown in Figure 7. At a content of 0.1%, the interfacial bonding strength between modified PVA fibers and polyurethane matrix was significantly improved, and both mechanical properties and thermal aging resistance of the composite materials were notably enhanced.

#### 3.3.3. Polymer Blending Modification

**Figure 8:**
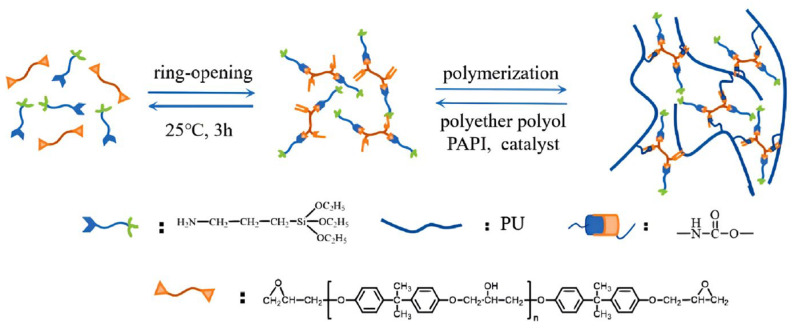
Mechanism of silane coupling agent bridging between epoxy resin and polyurethane. Reprinted with permission from ref. [73].

Polymer blending modification achieves comprehensive performance advantages of different polymers by introducing other polymers to form interpenetrating network (IPN) structures or blending systems. Xing Guolin et al. [71] prepared ring-opening epoxy resin (EP-OH)/polyurethane (PU) simultaneous interpenetrating polymer networks (IPNs) grouting materials by grafting polyurethane with ring-opening synthesized epoxy resin prepolymers. The partially ring-opened epoxy resin effectively improved the mechanical properties of the material, with tensile strength reaching 74.3 MPa, impact strength of 10.3 kJ/m^2^, and maximum adhesive strength of 10.4 MPa. The maximum heat accumulation temperature of the composite grouting material decreased from 142 °C to 118 °C. Xiaodan Li et al. [72] developed polyurethane/epoxy grouting materials with an initial viscosity reduced to below 200 mPa·s. Compared with pure epoxy resin, tensile strength, compressive strength, and impact strength were improved by 87%, 20%, and 50%, respectively, while volume shrinkage was significantly reduced. Junjie Wang et al. [73] synthesized organosilicon-modified epoxy resin using silane coupling agent-modified epoxy resin. The introduction of alkoxysilane groups increased the flexibility of polyurethane molecular chains, reducing slurry viscosity by 48.7% and improving solidified body fracture toughness by 28.7%, with the modification mechanism shown in Figure 8. Zhengpeng Yang et al. [74] prepared flexible polyurethane/water glass grouting materials with a three-dimensional interpenetrating network structure through carbon double bond polymerization and multi-component synergistic action, optimizing the ratio of N-methylol acrylamide, butenediol, water glass, and prepolymers, significantly improving the brittleness problem of traditional polyurethane grouting materials.

#### 3.3.4. Filler Surface Modification

**Figure 9:**
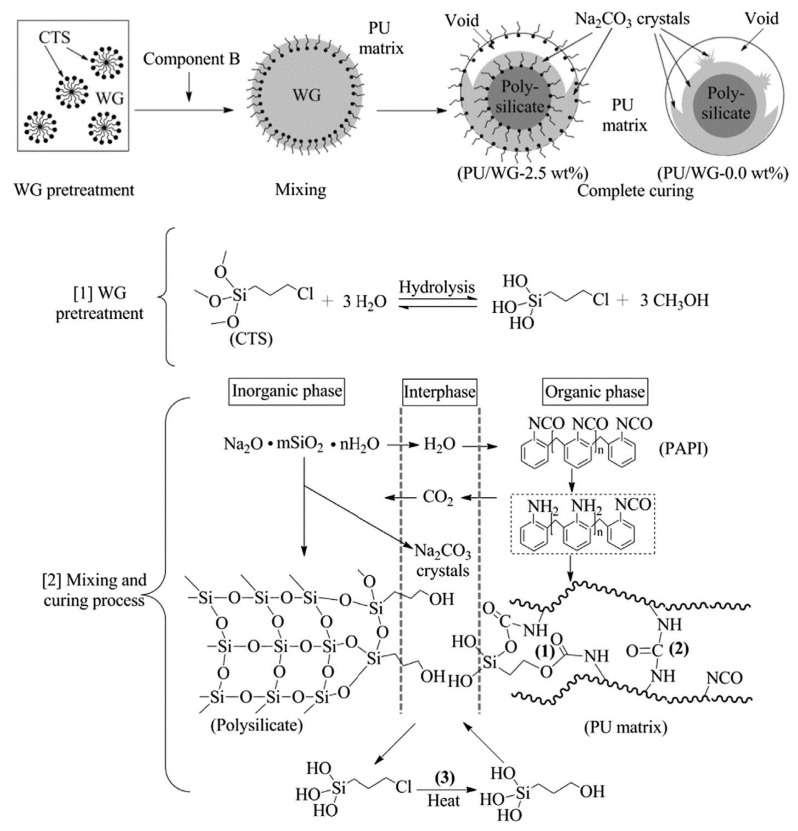
Mechanism of silane coupling agent modification for inorganic filler treatment. Reprinted with permission from ref. [76].

Filler surface modification improves the mechanical properties of composite materials by surface treating inorganic fillers to enhance interfacial compatibility with the polyurethane matrix. Dongdong Xu et al. [75] used an aluminate coupling agent for surface modification of BaSO_4_. The modified BaSO_4_ exhibited excellent hydrophobic properties (contact angle 133.6°), effectively reducing agglomeration and enhancing dispersion. Zhenglong He et al. [76] improved water glass (WG) surface properties using silane coupling agent 3-chloropropyltrimethoxysilane (CTS). By improving the uniform distribution of polysilicate particles in PU matrix and increasing crosslinking density, the compressive properties of PU/WG composites were improved by 11.65–40.65%, fracture toughness by 9.68%, fracture energy by 21.33%, and flexural strength and flexural modulus by 6.60% and 15.85%, respectively, with the modification mechanism shown in Figure 9.

### 3.4. Environmental Adaptability Improvement

In complex and extreme environments such as underground tunneling, marine engineering, and mining applications, polyurethane grouting materials often face severe challenges [77]. For example, dispersion and erosion issues in dynamic water environments, corrosion from various chemical media, and performance degradation under temperature and humidity variations [78]. These environmental factors may lead to grouting failure and structural safety risks, seriously affecting engineering quality and safety [79]. Therefore, developing corresponding modification technologies to address these environmental challenges is crucial for ensuring the stable performance of polyurethane grouting materials under harsh working conditions.

#### 3.4.1. Improvement of Water Dispersion Resistance

**Figure 10:**
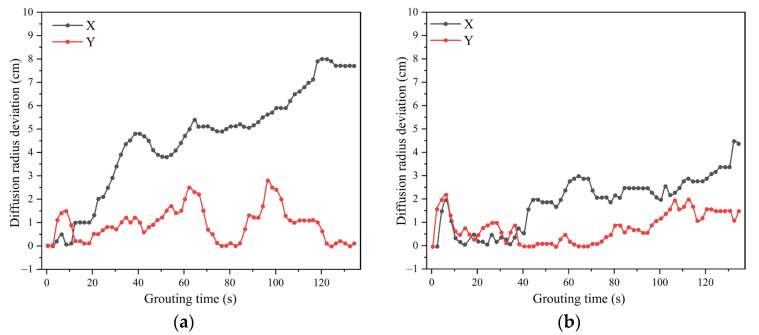
Diffusion radius deviation of polyurethane grouting materials in dynamic water conditions: (**a**) unmodified WPU, (**b**) HPMC-modified WPU. Data adapted from ref. [81].

Traditional polyurethane grouting materials are easily washed away and diluted in dynamic water environments, resulting in reduced solidification strength and poor plugging effectiveness. Chen Daqing et al. [80] used a combination of polyether polyol and polyester polyol as the polyol system, with polyvinyl acetate (PVAc) propylene carbonate solution as a viscosity-enhancing resin, to prepare water dispersion-resistant PU foam grouting materials. The PVAc enhances water dispersion resistance by increasing slurry viscosity and forming a hydrophobic membrane on the surface when the water-soluble propylene carbonate is washed away, while the insoluble PVAc precipitates to create a protective barrier. These materials demonstrated good compressive strength, durability, and water dispersion resistance, making them suitable for water plugging and reinforcement treatment in dynamic water environments. Xiaofan Liu et al. [81] modified water-soluble polyurethane by adding hydroxypropyl methylcellulose (HPMC), improving densification with heterogeneity reduced by 50.4% and bonding strength increased by 153%. In dynamic water grouting experiments, the diffusion radius deviation decreased from 7.7 cm to 4.39 cm, significantly improving the water dispersion resistance of the modified material, with the dynamic water grouting diffusion performance shown in Figure 10. The mechanism by which HPMC improves water dispersion resistance is that HPMC can absorb large amounts of water and retain them without dissolving, while HPMC and polyurethane molecules interpenetrate through hydrogen bonding between polar groups to form a three-dimensional interpenetrating network polymer (IPN). This enhanced network increases cohesion and mechanical properties of the slurry, and the modified system forms a protective gel barrier at the periphery that prevents excessive water penetration while maintaining good flowability. Yang Zhu et al. [82] used phosphoric acid as a retarder, ethyl acetate as a solvent, and PM-33 as a catalyst, modified with 4% nano-silica. The nano-silica modification enhances water dispersion resistance through its high specific surface area, providing numerous nucleation sites for polymer chain arrangement. Silanol groups (-Si-OH) on nanoparticle surfaces chemically bond with isocyanate groups, forming stable Si-O-C linkages that improve interfacial compatibility and create tortuous diffusion paths that impede water penetration. This approach produced grouting materials with a high water swelling ratio and environmentally friendly, non-toxic reaction processes and products, providing a new single-component underwater rapid-setting expanding polyurethane grouting material for grouting projects under complex hydrogeological conditions.

#### 3.4.2. Enhancement in Corrosion Resistance

Polyurethane grouting materials may come into contact with various chemical media during service, such as acids, bases, salts, and other corrosive substances. Zhenyang Wang et al. [83] investigated the effects of different chemical corrosion environments on the mechanical properties of polyurethane grouting materials by immersing PU materials of different densities in water, H_2_SO_4_, HCl, and NaOH solutions, and conducting P-wave velocity tests and uniaxial compression tests. The research found that water had minimal impact on the materials, while acidic and alkaline environments significantly reduced material strength. The maximum decreases in yield strength of PU samples after corrosion by H_2_SO_4_, HCl, and NaOH solutions were approximately 32.8%, 31.3%, and 21.4%, respectively. Shuchen Li et al. [43] studied the adaptability of polyurethane/water glass materials under seawater conditions and found that seawater corrosion reduced the peak strength and strain of the materials by 10.9% and 19.6%, respectively. However, the minimum strength and strain remained at 37.7 MPa and 26.9%, respectively, indicating high fatigue resistance and making them suitable for subsea tunnel reinforcement grouting.

#### 3.4.3. Improvement of Temperature and Humidity Adaptability

**Figure 11:**
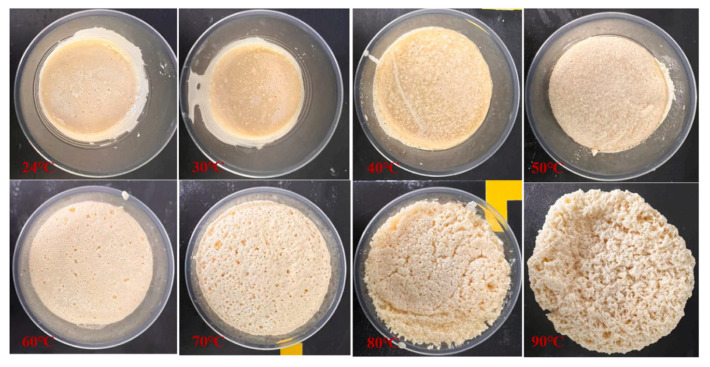
Curing process of polyurethane grouting materials at different raw material temperatures. Reprinted with permission from ref. [86].

Temperature and humidity are important environmental factors affecting the performance of polyurethane grouting materials. High temperatures accelerate material aging, low temperatures affect reaction rates, while humidity influences the bonding performance and mechanical properties of materials. Guo Xiaoxiong et al. [84] investigated the effects of environmental temperature on the mechanical properties of silicate-modified polyurethane grouting materials. At 25 °C, the compressive strength, tensile strength, compressive elastic modulus, and tensile elastic modulus of the slurry solidified body reached 48.16, 15.47, 507.47, and 520.10 MPa, respectively. When the environmental temperature increased to 90 °C, these values decreased to 38.13, 9.63, 387.57, and 410.84 MPa, respectively. The temperature increase disrupted the chemical reaction balance between the two components, reducing the crosslinking degree of the organic phase and the densification of the solidified body. Gao Shuang et al. [85] found through three-point bending tests that as environmental temperature increased, the polyurethane matrix underwent degradation, leading to decreased load-bearing capacity, fracture toughness, and fracture energy, while bending strain increased. Humid environments slightly reduced the fracture toughness and fracture energy of the material. Qinhao Huang et al. [86] systematically investigated the effects of raw material temperature on the performance of silicate-modified polyurethane grouting materials. By considering the direct mass ratio of raw materials and environmental factors during the curing process, they established a PU/WG curing time variation model. The research found that when raw material temperature increased from 24 °C to 60 °C, the peak strength decreased from 41.65 MPa to 14.51 MPa, and the material exhibited comprehensive responses to environmental effects, weak formation effects, and basic characteristics under high-temperature conditions, with the curing process phenomena at different raw material temperatures shown in Figure 11. Mingtian Wang et al. [87] studied the durability performance and evolution mechanism of polyurethane-modified water glass grouting materials under hydrothermal aging conditions, finding that the aging process mainly proceeded slowly from outside to inside, and the material possessed good durability.

However, current research primarily focuses on individual temperature or humidity effects, with limited investigation into combined hydrothermal aging conditions that more accurately simulate field environments. The synergistic interactions between temperature and humidity can accelerate polymer degradation through coupled mechanisms, where elevated temperatures facilitate moisture diffusion while humidity promotes hydrolytic chain scission. Future research should prioritize comprehensive hydrothermal aging studies to establish performance prediction models and develop testing protocols for evaluating long-term durability under combined environmental stresses.

#### 3.4.4. Enhancement in Environmental Sustainability

With increasing environmental protection awareness, traditional polyurethane grouting materials present significant environmental challenges, including toxic emissions and non-biodegradable formulations. Bio-based raw materials for sustainable polyurethane include various renewable feedstocks such as vegetable oil-derived polyols (castor oil [88], soybean oil [89], rapeseed oil [90]), plant-based biomass polyols (lignin-based polyols [91], tannin derivatives [92]), and waste-derived polyols (crude glycerol [93]). These renewable materials have demonstrated successful applications in rigid foams [94], flexible foams [95], coatings [96], and adhesives [97].

**Figure 12:**
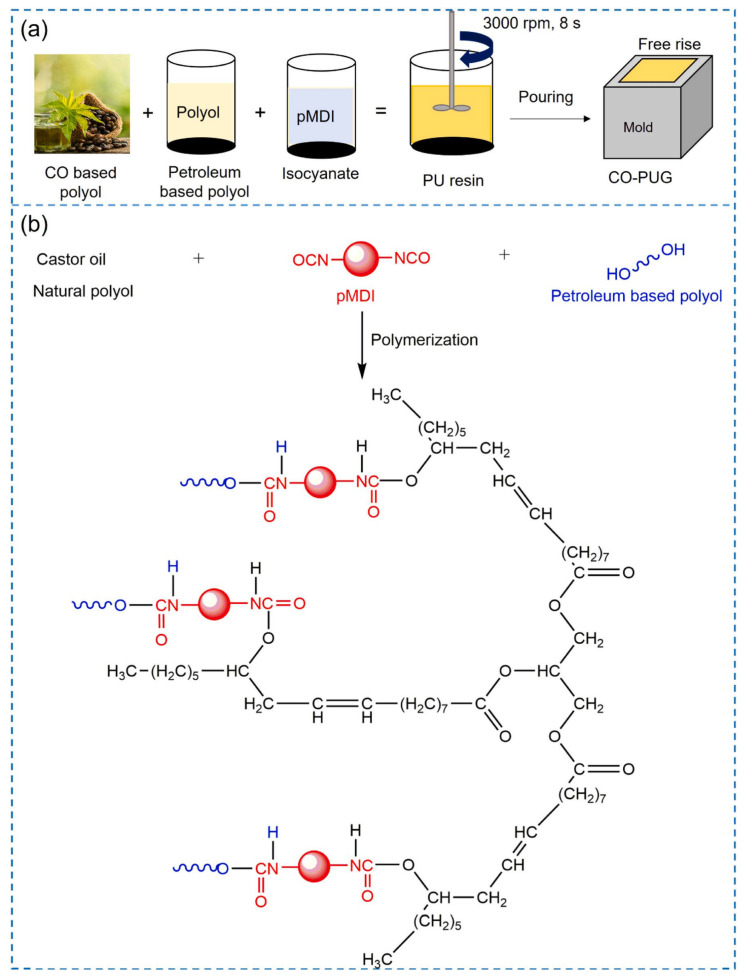
Preparation process and reaction scheme of castor oil-based polyurethane grouting materials: (**a**) preparation process, (**b**) reaction scheme. Reprinted with permission from ref. [98].

Abdultawab et al. [98] developed castor oil-based polyurethane grouts (CO-PUG) by partially replacing petroleum-based polyols with castor oil at varying concentrations (0–80 wt%). The research demonstrated that CO-PUG80 achieved remarkable performance improvements, with compressive strength increasing by 730% compared to conventional PUG, reaching 1085.01 kPa. The material exhibited excellent water resistance with a low permeability coefficient of 2.624 × 10^−8^ m/s and enhanced shear properties, with the shear resistance angle reaching 47.35° and cohesion improved by 260%. The closed-cell structure substantially reduced water absorption, making it particularly suitable for grouting applications in dynamic water environments, as shown in Figure 12. Chen et al. [99] synthesized environmentally benign bio-based waterborne polyurethane (WPU) using soybean oil as the initial raw material through epoxidation, hydroxylation, and stepwise polymerization processes. The bio-based WPU was further enhanced through composite modification combining γ-aminopropyltriethoxysilane (APTES) chemical crosslinking with nano-SiO_2_ physical reinforcement. The modified bio-based WPU demonstrated significant improvements in tensile strength (increased to 3.78 MPa), water resistance (water absorption reduced to less than 1% after 28 days), and thermal stability (glass transition temperature increased to 7.61 °C). The material achieved excellent bonding performance with cementitious materials, with interfacial bonding strength increased by 206.56% to reach 1.87 MPa, making it promising for concrete protection applications.

These advances demonstrate the feasibility of developing sustainable polyurethane grouting materials while maintaining performance characteristics. However, bio-based modifications in grouting applications remain significantly limited, with most research concentrated in coatings, adhesives, and foam materials. The grouting field continues to rely predominantly on petroleum-based systems, creating a notable research gap in bio-based grouting technologies. Future development should prioritize bio-based polyurethane grouting materials that can achieve comparable performance while providing environmental benefits.

## 4. Performance Characterization Methods of Polyurethane Grouting Materials

The performance quality of polyurethane grouting materials directly determines their engineering application effectiveness and service life. With the continuous development of modification technologies, characterization methods for material performance are also constantly being deepened and improved. To comprehensively evaluate the overall performance of modified polyurethane grouting materials, it is necessary to establish a multi-dimensional characterization system covering slurry properties, solidified body performance, microstructural characteristics, and environmental adaptability. Table 3 systematically summarizes the main performance indicators, testing methods, and testing purposes of modified polyurethane grouting materials, including six aspects: slurry properties, solidified body performance, microstructure, thermal properties and flame retardancy, diffusion grouting performance, and environmental adaptability.

It is worth noting that complex interrelationships exist between these performance indicators. For example, reducing reaction temperature often prolongs gel time. Fan et al. [50] demonstrated that microencapsulated phase change materials reduced maximum curing temperature from ~140 °C to 98.7 °C (30% reduction) while extending curing time to 235 s; increasing density can enhance mechanical properties but reduce flowability. Wang et al. [44] showed that foaming materials (density 0.1–0.8 g/cm^3^) exhibit initial viscosities of 200–800 mPa·s with high expansion ratios, while elastomer types (density 1.0–1.4 g/cm^3^) have higher viscosities of 800–2000 mPa·s but superior load-bearing capacity. Therefore, comprehensive trade-offs and optimized design are required in practical applications according to specific working conditions.

Additionally, when multiple modification technologies are applied simultaneously, synergistic or antagonistic interaction effects may occur beyond simple additive responses. Han et al. [52] demonstrated that the combination of expanded graphite (EG) and dimethyl methylphosphonate (DMMP) achieved synergistic effects, reducing maximum reaction temperature by 36.1% while simultaneously decreasing viscosity by 33.2%, indicating that thermal conductivity enhancement and chemical cooling mechanisms can work cooperatively. Similarly, Zhang et al. [59] found that nano-layered double hydroxide (LDH) combined with fly ash produced both temperature reduction (26.7 °C decrease) and improved flame retardancy, suggesting positive interactions between different modification approaches. Understanding these modification interaction effects is crucial for developing optimized multi-functional formulations and avoiding potential antagonistic combinations that may compromise overall performance.

The performance characterization framework in Table 3 establishes clear relationships between specific modification technologies and their target performance outcomes. Temperature reduction modifications primarily affect thermal-related indicators (maximum reaction temperature, thermal decomposition temperature), flame retardant modifications directly target fire safety parameters (LOI, heat release rate), mechanical enhancement modifications focus on strength-related properties (compressive strength, fracture toughness), while environmental adaptability modifications address durability indicators (corrosion resistance, water dispersion resistance).

## 5. Engineering Applications of Polyurethane Grouting Materials

Polyurethane grouting materials, as high-performance engineering materials, have been widely used in underground engineering, infrastructure, and environmental remediation fields. According to their main functions in engineering, the applications can be classified into three major categories: grouting reinforcement, grouting water plugging, and grouting repair.

### 5.1. Grouting Reinforcement

**Figure 13:**
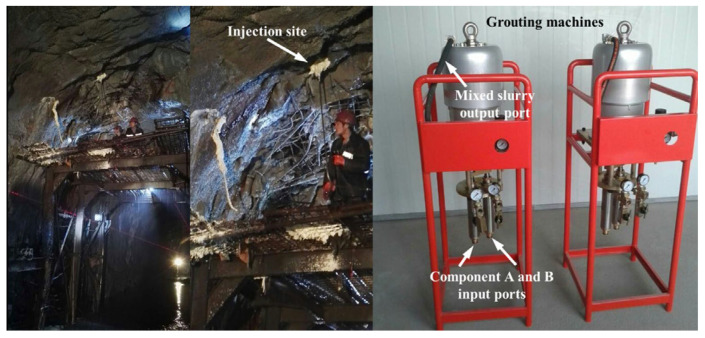
Field application of polyurethane grouting materials in tunnel roof reinforcement. Reprinted with permission from ref. [76].

Compared with particulate slurries, polyurethane grouting materials, as chemical grouts, demonstrate excellent engineering performance in grouting reinforcement applications. They possess better permeability and reactive curing characteristics, enabling them to penetrate into fine pores and form solid consolidated bodies through chemical reactions. Chaojie Wang et al. [107] developed permeable polyurethane materials that were successfully applied to low-permeability silty soil foundation reinforcement. The study was conducted on silty soil from a foundation pit project in Zhengzhou, China, characterized by low permeability with 87.32% of particles smaller than 0.075 mm. The treated silty soil achieved compressive strength of up to 5 MPa, which proved more effective than traditional grouting materials such as cement and water glass in improving the mechanical properties of low-permeability silty soil. In the field of wind turbine foundation reinforcement, Pan Wang et al. [108] developed a new polyurethane elastomer grouting material that exhibited outstanding performance. This material could achieve compressive strains exceeding 45% and tensile strains exceeding 40% before ultimate failure, demonstrating stronger deformation resistance compared to traditional cement-based grouting materials. Zhong Xinhua et al. [109] developed modified polyurethane grouting materials with characteristics of low viscosity, controllable curing time, low volume change, and low water absorption for high-speed railway subgrade reinforcement requirements. The material was successfully applied in a coastal railway subgrade reinforcement project in Southeast China in November 2014, with one-year settlement monitoring confirming good application performance. Engineering applications proved that this material could meet the practical requirements of high-speed railway subgrade reinforcement, effectively controlling subgrade settlement while ensuring structural stability. Typical applications of polyurethane grouting materials in engineering projects, such as tunnel roof reinforcement, are shown in Figure 13.

### 5.2. Grouting Water Plugging

Polyurethane grouting materials demonstrate excellent engineering performance in dynamic water sealing applications. Compared with traditional grouting materials, polyurethane materials can rapidly react and cure in dynamic water environments, expanding upon contact with water to form dense sealing bodies, particularly playing an important role in emergency water plugging under complex hydrogeological conditions. Tao Weiming et al. [110] addressed the water inrush problem in water-rich formations of the Baoshan Tunnel on the Dali–Ruili Railway, which traverses five fault fracture zones with maximum confined water flow rates reaching 15 L/s. They developed polyurethane composite grouting materials and successfully applied them to field treatment under grouting pressures of 1.0–2.0 MPa. The material employed organic/inorganic hybridization technology, achieving retention rates exceeding 90% in gravel formations under dynamic water conditions with flow velocities up to 1.5 m/s. After grouting, no water seepage was observed during excavation, and long-term monitoring over several months confirmed excellent sealing effects with permeability coefficients maintained at 3.068 × 10^−7^ cm/s. Song Xiangshuai et al. [111] addressed the water inrush problem in foundation pits with strongly developed water-rich karst formations at Kuichong Station of the Shenzhen–Huizhou Intercity Railway Dapeng Branch. They adopted a polyurethane grouting process involving “identifying seepage paths–cutting off hydraulic supply–reducing external hydraulic supply–reducing groundwater flow velocity and pressure,” with polyurethane injection flow rates of 6–12 L/min under groundwater flow velocities not exceeding 0.4 m/s. The treatment successfully reduced water inflow from 15 m^3^/h to 0.5 m^3^/h and completed emergency water sealing within 24 h, ensuring the construction safety of the station foundation pit with underground diaphragm walls spanning existing large karst collapse areas of unknown depth. Sun Qihao et al. [112,113] investigated the water plugging mechanism of polyurethane in shield tunnel leakage through model experiments conducted under water pressures ranging from 5.26 to 21.07 kPa and hydraulic gradients of 0.28–3.60. They found that the plugging process can be divided into three stages: polyurethane reaction, releasing gas to resist seepage water pressure, polyurethane curing to seal leakage channels, and complete polyurethane curing, causing external load redistribution. Testing was performed over 2–4 h periods with polyurethane gel times ranging from 15–240 min, depending on formulation, with better grouting plugging effects observed in silty soil formations. Pengcheng Wang et al. [114] conducted comparative studies of composite grouting technologies using foam polyurethane/water glass and reinforced polyurethane/water glass materials. The experiments were conducted under controlled conditions with a constant water pressure of 0.2 MPa maintained through the chamber, and a grouting flow rate of 30 mL/s. The test system included a transparent grouting chamber (150 mm diameter × 1500 mm length) with six pressure gauges installed at different locations to monitor pressure changes during grouting. The experimental procedure involved three phases: water injection (with pressure increased to 1 MPa for tightness testing), foam grouting (stopped when grouting volume reached 4 L), and reinforcement grouting (stopped when grouting pressure exceeded 1 MPa). They found that foam grouting can block water flow and pressure transmission, while reinforced grouting improves material strength, and recommended adopting a zoned grouting strategy of foam first, followed by reinforcement.

### 5.3. Grouting Lifting

**Figure 14:**
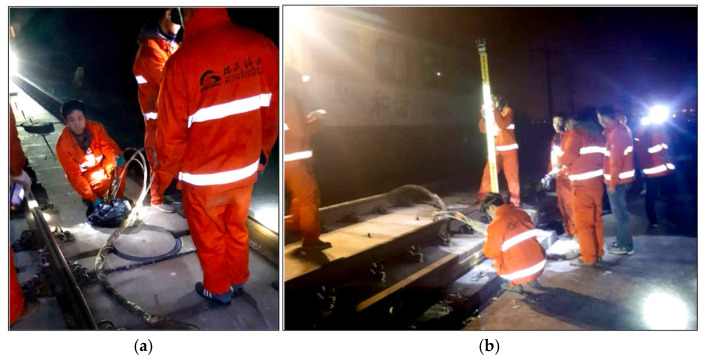
Field polyurethane grouting lifting process: (**a**) polyurethane injection and (**b**) rail surface elevation monitoring. Reprinted with permission from ref. [115].

Polyurethane grouting materials, due to their controllable expansion characteristics and excellent mechanical properties, can achieve precise lifting effects, particularly excelling in track settlement lifting and repair applications. Xuecheng Bian et al. [115] addressed the uneven settlement problem of ballastless tracks on high-speed railway lines along China’s east coast by employing polyurethane grouting lifting technology to repair and treat lines with 8 mm settlement over a 120 m range. The measurement system utilized LVDTs installed at track centers and corners for real-time displacement monitoring during the grouting process. Field application results showed that this technology successfully controlled the maximum uneven settlement from 8.0 mm to 1.7 mm, significantly improving track geometry. Continuous monitoring data for 126 days after construction showed that the track elevation remained stable with no further settlement, with the field grouting lifting construction process shown in Figure 14. Zhang Zhiyuan et al. [116] conducted optimization research on polyurethane lifting technology for subgrade mud pumping of CRTS I ballastless tracks on the Shanghai–Nanjing Intercity Railway. Using water-insensitive modified polyurethane grouting materials combined with multi-point coordinated multi-round grouting lifting processes, they could complete deep drainage reinforcement and lifting of water-rich settlement subgrade within a 20 m range during a single maintenance window, achieving lifting precision of 0.1 mm and effectively solving problems such as uncontrollable deformation and water sensitivity of traditional polyurethane materials. The remarkable 0.1 mm precision was achieved through rigorous pre-construction equipment calibration and material pre-sampling procedures, and careful control of environmental factors, including water-rich conditions and temperature variations that could affect material reaction rates and measurement accuracy during the limited maintenance window.

## 6. Conclusions

Modified polyurethane grouting materials have achieved significant progress in temperature reduction modification, flame retardant modification, mechanical enhancement, and environmental adaptability, effectively solving key technical problems of traditional polyurethane grouting materials, such as high reaction heat release, high brittleness, and poor flame retardancy. They have demonstrated excellent application effects in engineering fields, including grouting reinforcement, grouting water plugging, and grouting lifting.

Despite extensive research efforts, several critical gaps remain in the current literature. Most existing studies focus on single modification approaches without a systematic summary of multi-performance trade-offs and synergistic effects. In-depth analysis regarding the relationship between modification mechanisms and performance outcomes remains limited. Additionally, insufficient attention has been paid to long-term durability, environmental impact assessment, and economic feasibility analysis. This review addresses these gaps through the following aspects: (1) establishing a comprehensive four-category classification system for modification technologies of polyurethane grouting materials; (2) systematically analyzing the synergistic or antagonistic interaction effects between different modification approaches; (3) constructing a complete evaluation framework from performance characterization to engineering applications, covering multi-dimensional characterization methods including slurry properties, solidified body performance, microstructure, as well as practical application analysis in three major engineering fields: reinforcement, water plugging, and lifting.

## 7. Future Perspectives

With the increasing use of chemical grouting materials, traditional engineering applications still mainly rely on conventional materials, with insufficient promotion and application of modified materials. Research has shown that adding cement or other cementitious materials to polyurethane can not only improve the strength and durability of composite materials but also effectively reduce the cost of grouting materials [44,117]. Cement-based materials typically cost 2–5 times less than polyurethane grouting materials. Future development should focus on achieving cost reduction through composite modification technologies and process optimization, establishing standardized performance evaluation systems, and promoting the transformation of modified polyurethane grouting materials from laboratory research to large-scale engineering applications.

Current modification technologies mainly target single performance issues, but different modification methods exhibit mutual constraints. For example, temperature reduction modification tends to prolong curing time, affecting construction efficiency; flame retardant modification often reduces material strength; mechanical enhancement modification increases cost and process complexity. These problems limit the practical application of modified materials [118]. Future research needs to develop synergistic modification technologies to resolve performance conflicts through optimized formulation combinations. The key is to comprehensively consider multiple indicators, including temperature reduction, flame retardancy, strength, environmental adaptability, water plugging performance, and durability, achieving coordinated development and balanced optimization of various performances through systematic design. Additionally, specialized modification technologies need to be developed for extreme conditions such as deep-sea high-pressure environments, high-temperature geothermal environments, and strongly corrosive environments, while developing rapid curing and controllable retardation technologies to meet the requirements of different construction conditions [119].

Although bio-based polyurethanes have been widely applied in multiple fields such as automotive, thermal insulation, biomedical, and shape memory applications, with their inherent physical properties enabling diverse application prospects in the forms of adhesives, coatings, and foams [120], they remain a blank field in actual grouting engineering with limited applications. Biomass resources such as vegetable oils, polysaccharides, and lignin have broad application prospects in bio-based polyurethane production, providing a technical foundation for the green development of grouting materials. Future research should strengthen studies on bio-based raw material substitution, solid waste resource utilization, and clean production processes to promote the development of grouting materials toward green and low-carbon directions [121].

## Figures and Tables

**Table 1:** Molecular structures and formulas of the main polyurethane grouting material components.

Items	Chemical Name	ID	Structural Formula	Molecular Formula
Isocyanate	2,4-Toluene diisocyanate	2,4-TDI	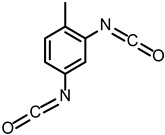	C_9_H_6_N_2_O_2_
	2,6-Toluene diisocyanate	2,6-TDI	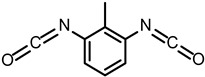	C_9_H_6_N_2_O_2_
	4,4′-Diphenylmethane diisocyanate	MDI	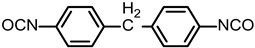	C_15_H_10_N_2_O_2_
Polyols	Polypropylene glycol	PPG	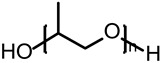	C_3n_H_6n+2_O_n+1_

**Table 3:** Main performance indicators and characterization methods of polyurethane grouting materials.

Equipment Type	Performance Indicator	Testing Method	Testing Purpose
Universal Testing Machine	Compressive strength [57,66]	Uniaxial compression test	Evaluate load-bearing capacity
	Tensile strength [71,73,75]	Uniaxial tensile test	Evaluate crack resistance
	Fracture toughness [76,85,100]	Three-point bending test	Evaluate fracture resistance
	Fatigue life [101,102]	Cyclic loading test	Predict service life
Viscometer/Rheometer	Initial viscosity [66,67,72,73]	Rotational viscometer	Evaluate construction injectability
	Gel time [66,67]	Cup tilting method/Rotational viscometer	Control grouting time
Thermal Analysis Equipment	Maximum reaction temperature [47,52,53,54,62]	Infrared thermometer	Evaluate safety
	Thermal decomposition temperature [55,62]	Thermogravimetric analyzer (TG)	Evaluate thermal stability
	Heat release rate [52,53]	Cone calorimeter	Evaluate combustion safety
	Limiting oxygen index [52,53,58,59,60]	Oxygen index apparatus	Evaluate combustion safety
Microscopy Equipment	Foam cell morphology [66,102,103]	Scanning electron microscopy (SEM)	Analyze structural characteristics
	Chemical structure [54,68]	Fourier transform infrared spectroscopy (FTIR)	Analyze the reaction mechanism
	Phase composition [54]	X-ray diffraction (XRD)	Determine phase structure
Physical Testing Equipment	Foaming ratio [66,67,69]	Density tester	Predict expansion performance
	Curing time [38,43]	Touch test method	Control construction time
Specialized Grouting Equipment	Diffusion radius [104,105]	Grouting test/Numerical simulation	Design grouting parameters
	Permeability coefficient [106]	Permeability test	Evaluate plugging effectiveness
Environmental Testing Equipment	Corrosion resistance [83,87]	Immersion test	Predict durability
	Water dispersion resistance [80,81]	Dynamic water erosion test	Evaluate dynamic water stability
	Temperature and humidity stability [84,85,87]	Aging test	Predict service life

## Data Availability

The original contributions presented in this study are included in the article. Further inquiries can be directed to the corresponding authors.

## Abbreviations

The following abbreviations are used in this manuscript:

PU	Polyurethane
MDI	4,4′-Diphenylmethane diisocyanate
TDI	Toluene diisocyanate
2,4-TDI	2,4-Toluene diisocyanate
2,6-TDI	2,6-Toluene diisocyanate
PPG	Polypropylene glycol
NCO	Isocyanate groups
WG	Water glass
MCG	Modified coal gangue powder
EG	Expanded graphite
DMMP	Dimethyl methylphosphonate
LDH	Layered double hydroxide
MEPCMs	Microencapsulated phase change materials
S-MPCM	Silica-based urea-formaldehyde heteroshell phase change microcapsules
HPMC	Hydroxypropyl methylcellulose
PVAc	Polyvinyl acetate
APTES	γ-aminopropyltriethoxysilane
CTS	3-chloropropyltrimethoxysilane
PVA	Polyvinyl alcohol
EP-OH	Ring-opening epoxy resin
IPN	Interpenetrating polymer networks
SEM	Scanning electron microscopy
FTIR	Fourier transform infrared spectroscopy
XRD	X-ray diffraction
TG	Thermogravimetric analyzer
LOI	Limiting oxygen index
UL-94	Underwriters Laboratories Standard 94
CRTS	China Railway Track System
WPU	Water-soluble polyurethane
